# Prize Essays

**Published:** 1851-10

**Authors:** 


*Prize Essays.*—We would call the attention of our readers to the card of the committee appointed by the American Medical Association to receive essays competing for prizes, as provided by the resolutions adopted at the last meeting. The card referred to may be found in the Eclectic department of the present No. of this Journal.

The plan adopted by the Association is to appoint a committee consisting of members residing in the same district, for the convenience of meeting to confer on the objects for which they were appointed. This accounts for the fact that all the members of the present committee reside at, or near Boston, Mass. It is proposed that the committee shall, on successive years, be located in different sections of the country.

